# Vowel perception in multilingual speakers: ERP evidence from Polish, English and Norwegian

**DOI:** 10.3389/fpsyg.2023.1270743

**Published:** 2023-10-12

**Authors:** Hanna Kędzierska, Karolina Rataj, Anna Balas, Zuzanna Cal, Chloe Castle, Magdalena Wrembel

**Affiliations:** ^1^Department of Contemporary English Language, Faculty of English, Adam Mickiewicz University, Poznań, Poland; ^2^Department of English and Comparative Linguistics, Institute of English Studies, University of Wrocław, Wrocław, Poland; ^3^Neuroscience of Language Laboratory, Department of Psycholinguistic Studies, Faculty of English, Adam Mickiewicz University, Poznań, Poland; ^4^Department of Language and Culture, UiT The Arctic University of Norway, Tromsø, Norway

**Keywords:** multilingualism, speech perception, third language (L3/L*n*), event-related potentials (ERP), mismatch negativity (MMN)

## Abstract

**Introduction:**

Research on Mismatch Negativity (MMN) in monolingual and bilingual speakers has shown significant differences in L1 versus L2 phonemic perception. In this study, we examined whether the MMN response is sensitive to the differences between L1, L2 and L3/L*n*.

**Methods:**

We compared bioelectrical brain activity in response to changes in pairs of vowels produced in three different languages. Specifically, multilingual participants listened to selected vowel contrasts in their L1 Polish, L2 English and L3/L*n* Norwegian presented within the passive-oddball paradigm.

**Results:**

Results revealed that the MMN was modulated by language: we observed significant differences between L2 English and L3/L*n* Norwegian as well as between L1 Polish and L3/L*n* Norwegian. For L3/L*n* Norwegian, the MMN response had a lower amplitude when compared with L2 English and L1 Polish.

**Discussion:**

Such findings suggest that foreign language status (i.e., L2 vs. L3/L*n*) modulates early auditory processing.

## 1. Introduction

Non-native phonemic perception is considered a vital component of successful language learning and has become a focus of scientific research. Due to global migration processes and the introduction of at least one foreign language at the early stages of education, multilingualism has become a norm rather than an exception in most European countries. Still, many issues related to the interaction of more than two languages in a single speaker are yet to be investigated, and, among them, those related to the phonemic perception mechanisms (Cabrelli and Wrembel, 2016). The problem, aside from associated theoretical implications, is particularly relevant from the point of view of language learners, who often aim at target-like non-native phoneme production. This intention is very strongly intertwined with their perception of foreign phonemes relative to native phonemes. Previous research has found significant neural differences in native as opposed to non-native phonemic perception, suggesting reduced phonemic discrimination mechanisms in the second language (L2) when compared with the first language (L1) (e.g., Jakoby et al., 2011; Song and Iverson, 2018; Liang and Chen, 2022). However, the listener’s auditory discrimination abilities in L3/L*n* remain largely understudied. While proficiency in L2 is generally considered an advantage in acquiring L3/L*n* phonologies, an ongoing scientific debate on multilingualism tends to highlight the complexity of multiple languages interacting in the same speaker (e.g., Wrembel, 2015; Slabakova, 2017; Westergaard et al., 2017; Wrembel and Cabrelli Amaro, 2018; Dziubalska-Kołaczyk and Wrembel, 2022). Investigating the neural pattern associated with trilingual (as opposed to bilingual) listeners could then greatly contribute to this debate. In the current study we investigated the mismatch negativity (MMN) event-related brain potentials (ERP) response to phonemic differences in participants whose L1 was Polish, L2 was English, and L3/L*n* – Norwegian. Given the previously observed discrepancies in phonemic discrimination in low versus high proficiency language learners (Liang and Chen, 2022), we also accounted for participants’ language proficiency and dominance.

The seminal study of Näätänen et al. (1997) revealed that listeners’ sensitivity to native phonemes can be indexed by the MMN component, thus beginning a series of studies focused on neural responses to phonemic stimuli. Näätänen et al. (1997) presented two groups of monolingual participants – Estonians and Finns – with vowel phonemes existent in both investigated languages (i.e., /e/ and /ö/) and the vowel /õ/ which has phonemic status in Estonian, but not in Finnish. The paradigm used in this and numerous other studies investigating phonological representations in the brain was the passive-oddball paradigm, where a sequence of frequently occurring *standard* stimuli is interrupted by the occasional appearance of a *deviant* stimulus. The use of the paradigm is frequently combined with the event-related brain potentials (ERP) technique, whose main advantage is its exceptionally high temporal resolution and hence high suitability for studying rapidly occurring cognitive processes, such as those related to language comprehension (Luck, 2005; Kaan, 2007; Cohen, 2014). Ideally, these processes may be further reflected in specific ERP components elicited as a reaction to the experimental manipulation and usually described on the basis of polarity, time of occurrence and scalp distribution.

In oddball tasks, the occurrence of the deviant is associated with the MMN response, i.e., a negative-going wave deflection with frontocentral distribution peaking around 150–250 milliseconds from the onset of the deviant (Kaan, 2007; Näätänen et al., 2007). The MMN, with its generators located in the auditory cortex (Alho et al., 1995), is believed to index auditory discrimination at the pre-attentional level. Thus, its elicitation does not require participants’ attention, which may be turned to other types of tasks, such as reading or watching a movie. The MMN is sometimes followed by the P300 component, i.e., a positive deflection observed at around 300 ms after change onset (Polich, 2012). P300 can be further divided into P3a and P3b sub-components, associated, respectively, with attentional switching and memory storage, which differ in terms of latency (with P3a occurring earlier) and distribution (with P3a being more anterior) (Roehm et al., 2007). Another component which has been demonstrated to follow the MMN is late discriminative negativity (LDN), i.e., a negativity observed over frontocentral sites at around 350–600 ms after change onset and typically associated with pre-attentive cognitive evaluation of the stimulus (Ceponiene et al., 1998; Jakoby et al., 2011; Liang and Chen, 2022). In Näätänen et al. (1997), the Estonian group showed an enhanced MMN response when compared with Finnish listeners if the deviant stimulus was /õ/, which has phonemic status only in Estonian. The finding suggested increased neural response to native phonemes and has consequently encouraged debate concerning phonemic discrimination in bilingual speakers.

Notably, further studies investigating phonological sensitivity in bilingual listeners have delivered divergent results, thus implying the importance of listener-oriented factors in the processing of non-native phonemic contrasts. Winkler et al. (1999) observed a similar MMN response to Finnish vowel contrasts in native speakers of Finnish and a group of Hungarians who were late learners of Finnish but who acquired the language to an advanced level in a naturalistic setting. However, in a similar study, Peltola et al. (2003) found a significant difference between native speakers of English and advanced students of English (native speakers of Finnish) who learnt English in a classroom setting. English vowel contrasts evoked lower MMN amplitudes in the latter group. This result seems to be further corroborated by Wottawa et al. (2022), who also found a diminished MMN response in proficient German learners who acquired German at school. The apparent discrepancy in the previously obtained results seems to indicate the importance of the learning context as a vital component of non-native phonemic perception. This hypothesis is additionally supported by the findings of Peltola et al. (2012) who observed that the MMN amplitude in dominant bilinguals depended on the language context of the experiment (i.e., the language used by the experimenter). In the current study, we focused on two foreign languages acquired in two different learning settings: most of our participants started learning English from age 10 onwards in the classroom setting and then migrated to Norway in adulthood, hence learning Norwegian at a later stage in life and in a much more naturalistic way.

Importantly, apart from the context of acquisition and the experimental setting *per se*, another factor which has been demonstrated to affect the pre-attentional phoneme discrimination in L2 is the level of proficiency. Liang and Chen (2022) found different neural responses in adult Mandarin learners of English with high and low proficiency levels. When processing non-native phonemic contrasts, bilinguals with high L2 proficiency showed the MMN response followed by late discriminative negativity (LDN). In contrast, participants with lower L2 proficiency showed the P3b component followed by the late positive component (LPC), i.e., a positivity observed in the parietal region between 250 and 600 ms (Liang and Chen, 2022). This result points to lower proficiency L2 learners’ reliance on memory resources in non-native phoneme discrimination. Furthermore, the study of Díaz et al. (2016) demonstrated that MMN was attenuated in poor L2 perceivers, i.e., a group of participants whose vowel contrasts perception was assessed as low in independent behavioral tasks (i.e., a categorization task, a word identification task and a lexical decision task; Díaz et al., 2016: 959). This finding suggests that individual speech-specific capabilities may be a source of variability in L2 phonemic learning.

The main objective of the current research is to shed more light on the perception of non-native phoneme contrasts, and more specifically, to determine whether such contrasts will be equally easy to detect in L2 and L3/L*n*. Testing trilingual listeners should expand the scope of research on both non-native phoneme perception and on multilingualism in general. This way we wished to we go beyond the bilingualism bias which in our opinion does not adequately reflect the current linguistic landscape. What is more, by testing trilingual participants who acquired their non-native languages through distinct modalities and exhibited diverse proficiencies, we wished to disentangle the divergent results of some of the previous studies on non-native phoneme perception in bilinguals.

Specifically, we investigated the perception of L1 Polish /ɨ/−/ɛ/, L2 English /ɪ/−/ʊ/ and L3 Norwegian /i/−/ʏ/ vowel pairs using the ERP technique in a passive oddball paradigm. It should be noted that there are significant differences between the sound systems of the three investigated languages, involving, among other phenomena, the vowel inventory density. While Polish has a fairly scarce vowel repertoire, with only six monophthongal vowels (Jassem, 2003), the vocalic inventories of English and Norwegian are richer with 12 and 18 monophthongal vowels, respectively, (Kristoffersen, 2000; Hawkins and Midgley, 2005; Bjelaković, 2017). The languages differ with respect to combination of lip-rounding with backness. All of them have front unrounded vowels and back rounded vowels, English and Norwegian have high central rounded vowels, whereas only Norwegian has front rounded vowels, which seem to be more marked (i.e., dispreferred among world languages; Maddieson, 2013). In the case of participants in the current study, the order of acquisition would then presume a gradual enlargement of the learners’ phonemic (and, specifically, vocalic) repertoire. The above-mentioned phonological differences between the three investigated languages motivated our decision to present vowel contrasts from each language independently, in separate experimental blocks (following previous researchers, e.g., Díaz et al., 2016; Liang and Chen, 2022).

For the sake of comparability of the influence of language status on the processing of native and non-native vowels, an ideal configuration of stimuli would involve the same standard stimulus in all the three languages, and deviants that would be equally distant in terms of all the features from the standard in all the three languages and at the same time these would need to be three different vowels. Such configurations are unattested in real languages; if phones are equidistant and differ with respect to the same features, they are the same sound. If we wanted to compare different vowels in the three languages, we needed to make compromises regarding the degree in which they differed.

Consequently, the choice of standard stimuli was motivated by the high degree of cross-linguistic similarity between the three standard sounds, i.e., the Polish /ɨ/, the English /ɪ/ and the Norwegian /i/ sound. The choice of deviants, on the other hand, was motivated by the systematic differences between the three investigated languages, which were briefly mentioned above. And thus, the Polish /ɨ/−/ɛ/ contrast is mainly manifested in height and is also existent in the other investigated languages. The English /ɪ/−/ʊ/ contrast is mainly manifested in backness and rounding and is also present in Norwegian, but absent in Polish, in which there is no near-high central rounded vowel. Finally, the Norwegian /i/−/ʏ/ contrast is mainly manifested in roundness and is absent in Polish and English, in which there are no front rounded vowels.

In the study we addressed the following research questions followed by associated predictions:

1. Will phonological contrasts be equally easy to detect and process in the native language (i.e., Polish) and non-native languages (i.e., English and Norwegian)?
   We predict the MMN effect to be larger in the native when compared with non-native vowel perception (Näätänen et al., 1997; Jakoby et al., 2011; Song and Iverson, 2018; Liang and Chen, 2022)
2. Will any significant distinctions emerge in L3/L*n* Norwegian as opposed to L2 English?
   The scale of the MMN effect in L2 when compared with L3/L*n* is difficult to predict due to the lack of previous studies which would focus on such a comparison. On the basis of previous L2 research, we can, however, tentatively assume that the MMN effect in L3/L*n* will be smaller relative to L1, and similar or smaller relative to L2. We can also predict the effect to be stronger in the more dominant and/or more proficient language.
3. What factors will play a crucial role in L2 and L3/L*n* phonological processing?
   Since studies in L2 phonemic perception point to the relevance of such factors as language proficiency (Liang and Chen, 2022), learning context (Peltola et al., 2003) and phonological aptitude (taken as a proxy indicator of the ability to discern between different sounds, see Díaz et al., 2016), we can also predict that these factors will affect the results of the current study on L2 as opposed to L3/L*n* phonemic perception.

## 2. Materials and methods

### 2.1. Participants

Twenty-one participants (mean age = 32.9, age range: 22–47, nine males) were recruited to take part in the study. They were all right-handed as assessed by the Edinburgh Handedness Inventory adapted from Oldfield (1971), with the mean laterality quotient (LQ) equal to 83.1% (range: 40.00%–100,00%, *SD* = 16.92%). All of the participants were originally from Poland and at the time of the study lived in Tromsø, Norway. Most of them were college graduates with an earned BA (*N* = 4), MA (*N* = 7) or PhD (*N* = 4) degree. Three participants were college students, and three reported high school as the highest completed level of education. According to self-reports, the ages of acquisition of the non-native languages was 9.48 years (range: 4–29, *SD* = 5.27) for L2 English and 27.33 years (range: 7^1^–43, *SD* = 8.21) for L3/L*n* Norwegian. For all the participants Polish was the only native language, and for all but two of them English was chronologically the first foreign language which they started learning at school or pre-school before puberty. The two participants started learning English at the ages of 15 (as the first foreign language) and of 29 (as the second foreign language, following Russian). The status of Norwegian differed more markedly among the participants: for various sub-groups, it was chronologically the third (*N* = 8), the fourth (*N* = 7), the fifth (*N* = 5), or even the sixth (*N* = 1) foreign language. The average length of residence in Norway equaled 7.79 years (range: 1–14, *SD* = 3.43).

The participants were asked to self-assess their knowledge of English and Norwegian in listening, speaking, reading and writing on a scale from 1 (very low) to 7 (proficient). In addition, their knowledge of the two investigated foreign languages was verified with the aid of two language proficiency tests taken immediately after the EEG session. The average score in the English proficiency test was 76.47% (range: 44.00–100.00%, *SD* = 15.85%), which would approximately correspond to the B2 level according to the CEFR proficiency scale. The average score in the Norwegian proficiency test was 58.65% (range: 22.22–94.44%, *SD* = 27.43%), which would approximately correspond to the A2 level according to the CEFR proficiency scale.

The summary of the participants’ biographic details and language proficiency can be found in Table 1. A more detailed summary of the language history questionnaire as well as proficiency tests results for individual participants are included in the Supplementary materials.

**Table 1:** The summary of the participants’ biographic details and language proficiency.

Participants	
*Biographic details*	
Age	M = 32.9, range: 22–47, SD = 7.4
Gender	12 females, 9 males
*Proficiency self-assessment*	
L1 Polish	M = 6.94, range: 5.75–7, SD = 0.28
L2 English	M = 5.76, range: 4.5–7, SD = 0.91
L3/L*n* Norwegian	M = 3.74, range: 1–5.75, SD = 1.76
*Proficiency tests results*	
L2 English	M = 76.47%, SD = 15.85%
L3/L*n* Norwegian	M = 58.65%, SD = 27.43%

None of the participants reported any neurological and psychiatric impairments nor any language-related issues (e.g., dyslexia, dysorthography). The participants signed an informed consent form before the experiment and received gift cards for their participation. Data from one participant (an L*n* speaker of Norwegian) was excluded from further analyzes due to technical issues.

### 2.2. Stimuli

Following Liang and Chen (2022), we used isolated vowels rather than vowels embedded in syllables within consonantal frameworks. Listeners are believed to process isolated vowels as speech thanks to the pre-attentive ability to extract the relevant F1/F2 formant ratio (Jakoby et al., 2011; Liang and Chen, 2022). Furthermore, using isolated vowels enabled us to investigate phonological contrast perception without any potential interference of co-articulation processes associated with syllable production, which are likely to be different in each of the three languages.

When it comes to the deviancy status of the selected vowels, in the Polish (L1) condition, the standard stimulus was the high central unrounded vowel /ɨ/ and the deviant stimulus was the high-mid front unrounded vowel /ɛ/ (as in the Polish words *byty* ‘beingpl’ and *bety* ‘bed linenpl’). In the English (L2) condition, the standard stimulus was the near-high front unrounded vowel /ɪ/ and the deviant stimulus was the near-high central slightly rounded vowel /ʊ/ (as in the English words *fit* and *foot* respectively). In the Norwegian (L3/L*n*) condition, the standard stimulus was high front unrounded vowel /i/ and the deviant stimulus was the near-high front weakly rounded vowel /ʏ/ (as in the Norwegian words *sin* ‘his_REFL_’ and *synd* ‘shame’ respectively). For the auditory stimuli, please visit our OSF repository: https://osf.io/2956a/?view_only=cf240fe1fab54b91a3aeab93c9e20423.

The vowels used in the current study were all synthesized with the aid of the PRAAT software (Boersma, 2001). Formant frequencies of Polish and English vowels were defined on the basis of the previous literature (Weckwerth and Balas, 2019 for Polish; Bjelaković, 2017 for English). Due to the lack of available literature, Norwegian vowels were generated based on the average values obtained from four native speakers of Norwegian (living in the Trondheim region). For all the synthesized stimuli the duration was 150 ms, the amplitude contour had a 3 ms linear onramp and 75 ms linear offramp, and the f0 trajectory had a steady linear fall from 140 Hz to 110 Hz. The formant values for each vowel as well as Euclidean distances between vowels used in the three language pairs are presented in Table 2. Our endeavors cannot be compared to the decisions made in previous studies, as their authors did not need to make choices concerning vowel pairs in three languages^2^.

**Table 2:** Summary of vowel formant frequencies used for stimuli synthesis (in Hz) and Euclidean distances between vowels (in Hz and Bark).

Vowel	F1	F2	F3	F4
Polish /ɨ/	468	1948	2821	3425
Polish /ɛ/	675	1916	2722	3441
English /ɪ/	394	1828	2882	3409
English /ʊ/	390	1345	2896	3413
Norwegian /i/	357	1917	2587	3505
Norwegian /ʏ/	313	2015	2708	3549

Euclidean distance	/ɨ/: /ɛ/	/ɪ/: /ʊ/	/i/: /ʏ/
F1-F2 (Hz)	209	483	107
F1-F2 (Bark)	2.05	4.42	1.06
F1-F2-F3 (Hz)	232	483	161
F1-F2-F3 (Bark)	2.27	4.42	1.59

### 2.3. Procedure

The participants were tested individually in a sound-attenuated room. At the beginning of each session, they were asked to fill in a language history questionnaire (based on Li et al., 2020) and a survey concerning hand dominance based on the Edinburgh Handedness Inventory (Oldfield, 1971). During the EEG session, participants were seated comfortably while watching a muted cartoon (*Bolek and Lolek*) without subtitles. The choice of a cartoon over other genres was motivated by the necessity to use the most engaging visual material possible which would direct the subjects’ attention away from the MMN-eliciting stimulus. Otherwise, attention-dependent ERP components might have overlapped with the MMN (Näätänen et al., 2007). Consequently, the participants were instructed to watch the movie carefully and attentively. They were also informed that they would be asked to answer a few questions about the content of the displayed story. The language of instruction was Polish.

The task sequence was controlled by a PC running Presentations software (Neurobehavioral Systems, http://www.neurobehavioralsystems.com). The sounds were presented binaurally through in-ear headphones. The loudness of the stimuli was kept constant across all participants. Each trial began with a phonetic sound for 150 ms, followed by a silence of 700–1,000 ms. The phoneme pairs were presented in three separate language blocks (i.e., Polish, English and Norwegian), the order of which was counterbalanced across participants. In each language block, 600 standards and 60 deviants were presented at an intensity of 75 dB, with a probability of 90.9% and 9.1%, respectively. The standard/deviant ratio was in accordance with previous studies for which the deviant probability varied between 6.7% (Díaz et al., 2016) and 16.7% (Liang and Chen, 2022). Deviant stimuli appeared in a pseudorandomized order, with a minimum of three preceding standard stimuli. Each experimental block was followed by a short break of approximately 3 min, during which time no stimuli were presented, and the participants continued watching the movie in silence. After the EEG session, the participants were asked to complete a short test concerning the content of the movie they had watched. The test consisted of 10 multiple choice questions (e.g., “Where did the boys hide after they broke the glass in the window? in barrels/in the closet/in the chimney”). The main purpose of the test was to help us determine whether the participants remained focused while watching the movie and whether the pre-attentive state for listening was successfully created.

Further, the participants took part in a gating task conducted in English with the aim of determining the potential individual differences in terms of speech-specific capabilities in a foreign language. We selected English as the language of the task, given that it was chronologically the first and more advanced foreign language spoken by the participants (which was further confirmed by the results of the proficiency tests and self-reports). While designing the task, we adapted the procedure used by Sebastián-Gallés and Soto-Faraco (1999) and later by Sebastian-Galles and Baus (2005) who applied a two-alternative forced choice test. The participants’ task was to identify the word whose fragment was presented via earphones by pressing “L” or “A” keys on the computer keyboard. The participants were also asked to assess how sure they were of their answer on a 7-point Likert scale. The experimental stimuli consisted of four monosyllabic word pairs including the /æ/−/ɛ/ contrast (i.e., *BAG-BEG*, *LAUGHED-LEFT*, *SHALL-SHELL*, *GAS-GUESS*). The alineation point (i.e., the point where the token words started to diverge) was determined on the basis of the visual inspection conducted with the aid of the PRAAT software (Boersma, 2001). This point was assumed to be “gate” 3. After the alineation point identification, the words were divided into other “gates” (i.e., fragments) by adding or subtracting 10 ms from the alineation point. Each member of the minimal pairs was presented two times, which resulted in 160 trials (4 pairs x 2 words x 10 “gates” x 2 presentations), with an optional break after 80 trials. The words were recorded by a native speaker of American English and presented at an intensity of 75 dB with the aid of the PsychoPy software (Peirce et al., 2019).

Finally, the participants were asked to complete two language proficiency tests: the Cambridge General English Assessment Test and the UiT Norwegian Placement Test. Thanks to this, we were able to adequately determine the participants’ level of proficiency in both foreign languages. A single experimental session lasted about 2.5–3 h, including the EEG preparation, EEG recordings and all the remaining tasks.

All procedures were accepted by the Ethics Committee for Research with Human Participants at Adam Mickiewicz University.

### 2.4. EEG data acquisition and analysis

The EEG signal was recorded using Brain Products LiveAmp acquisition device at a 500 Hz sampling rate from 32 active electrodes placed at the elastic cap according to the extended 10–20 convention. The ground was positioned at AFz. In addition, two electrodes were placed at the outer canthus of each eye (HEOG1 and HEOG2) and two were placed below and above the right eye (VEOG1 and VEOG2). The signal was referenced online to FCz, and later re-referenced offline to the average of right and left mastoid bones (approximated from TP7 and TP8). Electrode impedances were kept below 10 kΩ. The EEG data was processed with the aid of the Brain Vision Analyzer 2 software (Brain Products, Gilching).

At the first preprocessing state, the data were filtered offline with a 0.1–30 Hz band-pass filter. Then, a semi-automatic ICA ocular correction was performed and the signals were re-referenced. Epochs time-locked to the onset of each stimulus were extracted between −200 to 800 ms. Only the standard stimuli which immediately preceded a deviant stimulus were considered in the analysis; hence, the number of standard events and the number of deviant events were equal in each language (*N* = 60). Baseline correction was performed in reference to pre-stimulus activity (i.e., −200 to 0 ms). The next step of the analysis involved the semi-automatic Raw Data Inspection (maximal allowed voltage step: 50 μV/ms, maximal allowed difference of values in intervals: 200 μV/ms, minimal allowed amplitude: 100 μV, maximal allowed amplitude: −100 μV). Epochs contaminated by ocular or muscular artifacts were rejected from further analysis, which resulted in the exclusion of 1.57% of trials (1.08% for Polish standards, 1.67% for Polish deviants, 1.42% for English standards, 1.25% for English deviants, 2.08% for Norwegian standards and 1.92% for Norwegian deviants).

The separately averaged waveforms for the standard and the deviant stimuli were computed for each subject and the difference waveforms were then created by subtracting the standard response from the response to the deviant stimulus. Following Luck and Gaspelin (2017), we first averaged the waveforms elicited by standard and deviant stimuli across all the language conditions and defined the time windows used in our analysis based on the collapsed waveforms. This approach revealed an increased negativity in the 100–200 ms time window, which was followed by a late negativity in the 350–800 ms time window. Since use of the 100–200 ms time window is in accordance with Kujala and Näätänen (2003) and the 350–800 ms time window was also previously used in the literature (Di Dona et al., 2022), we used these time windows to measure the effects in the three language conditions separately. The analyzed region of interest was the frontal-central brain area (F3, Fz, F4, FC1, FCz, FC2, C3, Cz, C4), given that both the MMN and LDN effects are typically observed in this scalp site (Ceponiene et al., 1998; Kujala and Näätänen, 2003).

The statistical analysis of the results was conducted with the aid of the R software (R Core Team, 2012). More specifically, we used the lme4 package (Bates et al., 2012) to perform a linear mixed effects analysis of the relationship between the processed language and the status of the processed sound as standard or deviant. The procedure was carried out twice: in the earlier time window (i.e., 100–200 ms) for the MMN effect and in the later time window (i.e., 350–800 ms) for the LDN effect. Language (i.e., Polish, English and Norwegian) and sound (i.e., Standard or Deviant) were included in the model as fixed effects. As random effects, we included intercepts for participants and electrodes. The model was applied to data averaged across 60 trials in each of the language and sound conditions. Visual inspection of the residual plots did not reveal any obvious deviations from homoscedasticity or normality in either of the two analyzed time window data sets. *p* -values were obtained by likelihood ratio tests of the full model with the interaction effect in question against the model with two main effects.

In the following step, we compared effect sizes for significant effects observed in the lme analysis. For this reason, we calculated the difference wave (i.e., deviant minus standard) for each participant, individually in each electrode. Once again, we used the lme4 package (Bates et al., 2012) to perform a linear mixed effects analysis of the relationship between the processed language and the scale of the MMN and LDN effects. The procedure was also repeated: in the earlier time window (i.e., 100–200 ms) for the MMN effect and in the later time window (i.e., 350–800 ms) for the LDN effect. Language (i.e., Polish, English and Norwegian) was included in the model as fixed effects. As random effects, we included intercepts for participants. Visual inspection of residual plots did not reveal any obvious deviations from homoscedasticity or normality in either of the two analyzed time window data sets. *p* -values were obtained by likelihood ratio tests of the full model with the main effect of Language against the model with no main effects.

## 3. Results

### 3.1. Comprehension test results

The overall results of the movie comprehension test were very high, with the average of 93.81% correct responses (range: 70–100%, SD = 8.05%). This means that the participants focused on the movie rather than the experimental stimuli which were processed pre-attentively.

### 3.2. ERP results

**Figure 1:**
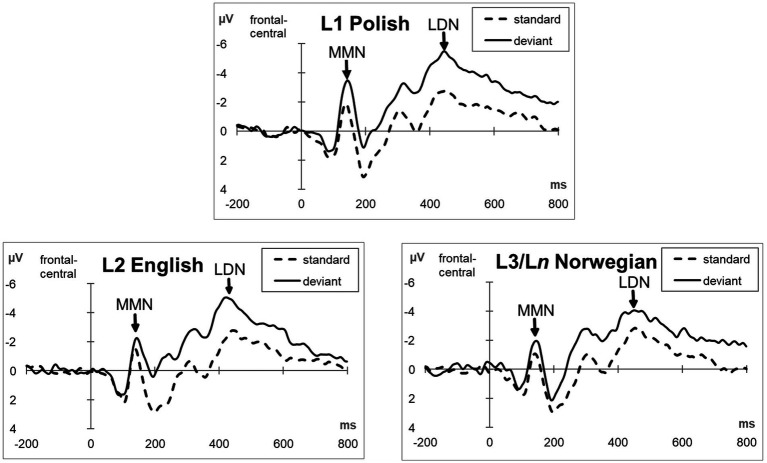
The grand average ERPs time-locked to the onset of the phoneme for the standard (dotted line) and deviant stimuli (solid line) elicited from nine representative electrodes (F3, Fz, F4, FC1, FCz, FC2, C3, Cz, C4) in the three investigated languages.

**Figure 2:**
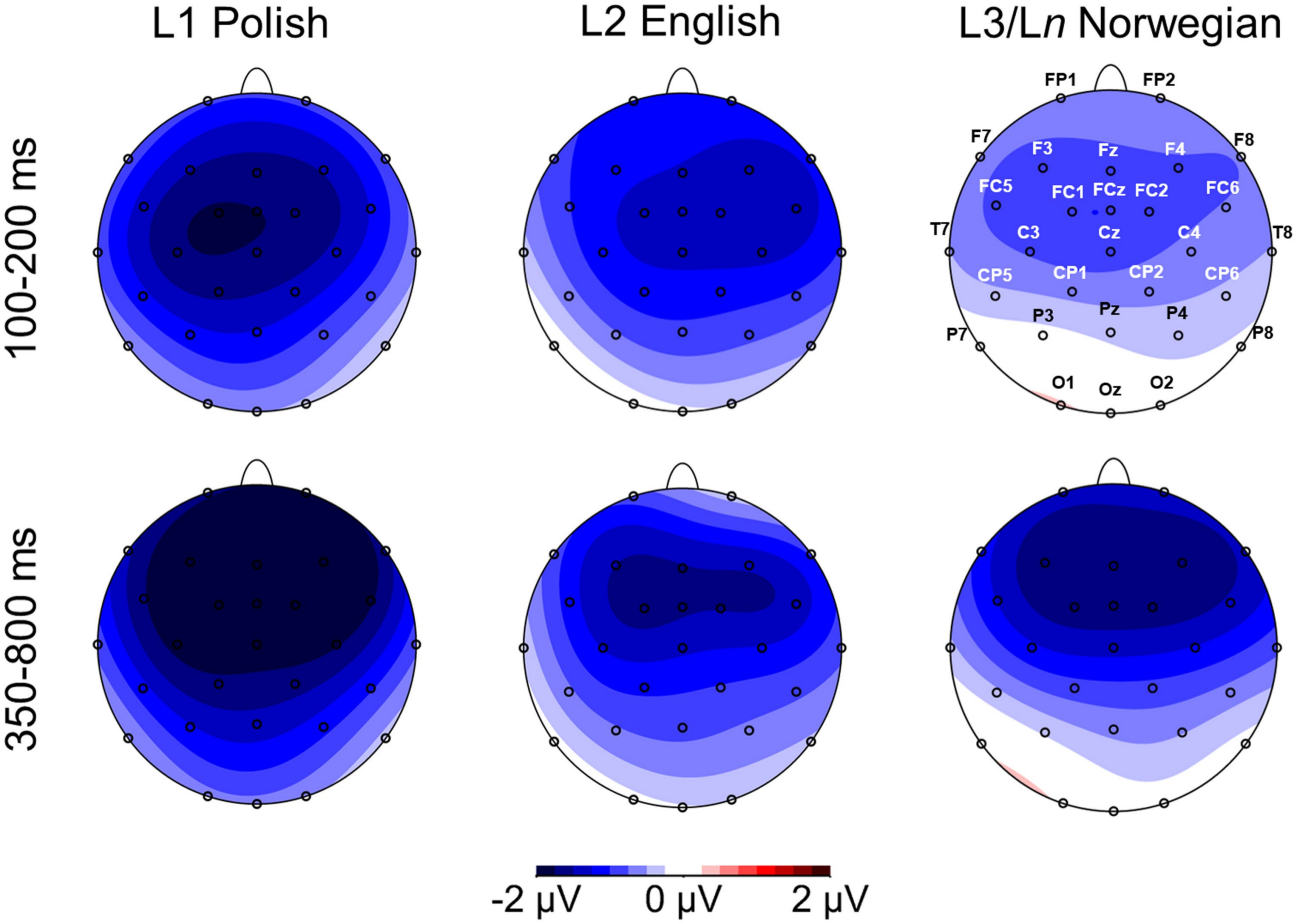
Topographic distribution of voltage differences between deviant and standard conditions in the three investigated languages in the 100-200 and 350-800 ms time windows.

The analysis revealed the MMN effect elicited as a reaction to deviant sounds when compared with standard sounds. The component was particularly pronounced over frontal-central scalp sites and had a peak at around 150 ms after the sound onset. The MMN effect was followed by the LDN, with a peak around 450 ms after the sound onset, which was also particularly well visible over frontal-central scalp sites. Figure 1 below presents grand average ERPs elicited from nine representative (F3, Fz, F4, FC1, FCz, FC2, C3, Cz, C4) electrodes in response to standard sounds (dotted lines) and deviant sounds (solid line) in the three investigated languages. Figure 2 presents a voltage difference map (deviant minus standard) in the analyzed time windows, i.e., 100–200 ms (for MMN) and 350–800 ms (for LDN).

Descriptive statistics for sound and language conditions in the two time windows of interest are presented in Table 3. Figures displaying mean amplitude values observed in each condition and each target language as well as mean amplitude differences in each target language are included in the Supplementary material.

**Table 3:** Descriptive statistics for the experimental conditions: standard/deviant and Polish/English/Norwegian.

	Emmean	SE	df	Lower.CL	Upper.CL
*Time window: 100–200 ms*					
L1 Polish					
Standard	0.47	0.381	23.3	−0.32	1.26
Deviant	−1.17	0.381	23.3	−1.95	−0.38
L2 English					
Standard	0.81	0.381	23.3	0.03	1.60
Deviant	−0.48	0.381	23.3	−1.27	0.31
L3/L*n* Norwegian					
Standard	0.78	0.381	23.3	−0.01	1.57
Deviant	−0.07	0.381	23.3	−0.86	0.72
*Time window: 350–800 ms*					
L1 Polish					
Standard	−1.36	0.28	28	−1.93	−0.79
Deviant	−3.42	0.28	28	−3.99	−2.85
L2 English					
Standard	−1.20	0.28	28	−1.77	−0.63
Deviant	−2.64	0.28	28	−3.21	−2.07
L3/L*n* Norwegian					
Standard	−1.11	0.28	28	−1.68	−0.54
Deviant	−2.64	0.28	28	−3.21	−2.08

#### 3.2.1. MMN

In the 100–200 ms time window, model comparison revealed a statistically significant interaction effect of language and sound (*χ*^2^ (2) = 21.554; *p* < 0.001). To further examine the significant interaction effect, Tukey based pairwise comparisons were performed, which revealed that in each language deviant sounds elicited significantly more negative amplitudes than standard sounds (*p* < 0.001) (see Table 4).

**Table 4:** Statistically significant pairwise comparisons between experimental conditions: standard/deviant and Polish/English/Norwegian.

Compared conditions	Estimate	SE	df	*t*.ratio	*p*-value
*Time window: 100–200 ms*					
L1 Polish					
contrast: deviant – standard	−1.63	0.119	1054	−13.755	<0.0001
L2 English					
contrast: deviant – standard	−1.30	0.118	1054	−10.938	<0.0001
L3/L*n* Norwegian					
contrast: deviant – standard	−0.86	0.119	1054	−7.176	<0.0001
*Time window: 350–800 ms*					
L1 Polish					
contrast: deviant – standard	−2.06	0.133	1057	−15.424	<0.0001
L2 English					
contrast: deviant – standard	−1.44	0.133	1057	−10.802	<0.0001
L3/L*n* Norwegian					
contrast: deviant – standard	−1.54	0.133	1057	−11.517	<0.0001

Degrees-of-freedom method: Kenward-Roger. *p*-value adjustment: Tukey method for comparing a family of 3 estimates.

Since we observed statistically significant differences in each analyzed language, we then calculated the difference wave (i.e., deviant minus standard) for each participant, individually in each electrode, and conducted a linear mixed effects analysis of the relationship between the processed language and the scale of the MMN effect (recall Section 2.4 for details). Descriptive statistics for language conditions are presented in Table 5. The deviant minus standard difference was the greatest in L1 Polish, a bit smaller in L2 English and the smallest in L3/L*n* Norwegian. In the 100–200 ms time window, model comparison revealed a statistically significant main effect of language (χ^2^(2) = 28.505; *p* < 0.001). Tukey based pairwise comparisons (see Table 6) revealed that the deviant minus standard difference was significantly higher in L1 Polish than in L3/L*n* Norwegian (Estimate = 0.775, *p* < 0.001) and significantly higher in L2 English than in L3/L*n* Norwegian (Estimate = 0.440, *p* < 0.01). The difference between L1 Polish and L2 English, however, was not statistically significant (Estimate = 0.336, *p* = 0.0521).

**Table 5:** Descriptive statistics for the MMN effect expressed in terms of the deviant minus standard difference in the three language conditions: Polish, English and Norwegian.

	Emmean	SE	df	Lower.CL	Upper.CL
*Time window: 100–200 ms*					
L1 Polish	−1.63	0.191	28.8	−2.02	−1.24
L2 English	−1.30	0.191	28.8	−1.69	−0.91
L3/L*n* Norwegian	−0.86	0.191	28.8	−1.25	−0.45
*Time window: 350–800 ms*					
L1 Polish	−2.06	0.309	23	−2.70	−1.42
L2 English	−1.44	0.309	23	−2.08	−0.8
L3/L*n* Norwegian	−1.54	0.309	23	−2.18	−0.898

**Table 6:** Pairwise comparisons for the MMN effect expressed in terms of the deviant minus standard difference in the three language conditions: Polish, English and Norwegian.

Compared conditions	Estimate	SE	df	*t*.ratio	*p*-value
*Time window: 100–200 ms*					
Contrast: English – Polish	0.336	0.144	518	2.334	0.0521
Contrast: Norwegian – Polish	0.775	0.144	518	5.387	<0.0001
Contrast: English – Norwegian	−0.440	0.144	518	−3.053	0.0067
*Time window: 350–800 ms*					
Contrast: English – Polish	0.617	0.161	518	3.824	<0.001
Contrast: Norwegian – Polish	0.522	0.161	518	3.233	<0.01
Contrast: English – Norwegian	0.095	0.161	518	0.591	0.825

Degrees-of-freedom method: Kenward-Roger; *p*-value adjustment: Tukey method for comparing a family of 3 estimates.

#### 3.2.2. LDN

The statistical analysis conducted in the 350–800 ms time window revealed a statistically significant language and sound interaction (*χ*^2^ (2) = 12.36; *p* < 0.01). Tukey based pairwise comparisons revealed that in each language deviant sounds elicited significantly more negative amplitudes than standard sounds (*p* < 0.001).

As in the case of the 100–200 ms time window, we observed a statistically significant negativity associated with the occurrence of a deviant in each investigated language. Consequently, we conducted an additional linear mixed effect analysis based on a model which included the deviant minus standard difference as a dependent variable. In the 350–800 ms time window this kind of analysis also revealed a statistically significant main effect of language (χ^2^(2) = 16.75; p < 0.001). Tukey based pairwise comparisons showed a statistically significant difference between L1 Polish and L2 English as well as between L1 Polish and L3/L*n* Norwegian, with the LDN effect significantly stronger in the case of Polish (Estimate = 0.617, *p* < 0.001 and Estimate = 0.522, *p* < 0.01 respectively). The difference between L2 English and L3/L*n* Norwegian was not statistically significant (Estimate = 0.095, *p* = 0.825).

### 3.3. Gating task results

In terms of the gating task, we calculated the mean accuracy for each participant as well as the mean ‘gate’ at which the words were recognized. While calculating the mean accuracy, we only took into account the answers which satisfied the following two criteria: (a) the decision concerning the selected word was not changed afterwards and (b) the level of confidence was assessed as at least 4 in a 7-point Likert scale. On average, the accuracy score equaled 78.87% (range: 50.00–100%, *SD* = 14.72%) and the words were recognized correctly after the eighth ‘gate’ (M = 8.23, range: 6.4–10, *SD* = 1.08).

To check whether the participants’ phonological aptitude (indexed by the results of the gating task) would influence the MMN or the LDN effect in English or Norwegian, we conducted several simple linear regression analyzes. None of them, however, yielded statistically significant results. They all included the overall gating accuracy or mean gates at which the words were recognized as predictor variables. The deviant minus standard value obtained for each participant in the respective language conditions (i.e., English or Norwegian) and time windows (i.e., 100–200 ms or 350–800 ms) were included as response variables. However, the amplitude of the investigated components was not significantly predicted by the participants’ gating accuracy (MMN in the English condition: *p* = 0.629, *R*^2^ = 0.013, LDN in the English condition: *p* = 0.949, *R*^2^ < 0.001; MMN in the Norwegian condition: *p* = 0.090, *R*^2^ = 0.151; LDN in the Norwegian condition: *p* = 0.969, *R*^2^ < 0.001) nor the mean of the “gates” at which the words were recognized (MMN in the English condition: *p* = 0.394, *R*^2^ = 0.041; LDN in the English condition: *p* = 0.870, *R*^2^ < 0.010; MMN in the Norwegian condition: *p* = 0.939, *R*^2^ < 0.001; LDN in the Norwegian condition: *p* = 0.114, *R*^2^ = 0.133).

Further, to verify whether the participants’ proficiency (self-assessed and further verified by two foreign language tests; recall Table 1), dominance (associated with the frequency of language use) or age of acquisition (self-reported in the LHQ) would affect the scale of the MMN or the LDN effect, we conducted additional linear mixed effect analyzes, independently for MMN and LDN. The first two analyzes included the self-reported proficiency scores as predictor variables and the deviant minus standard value obtained for each participant in the respective language conditions as a criterion variable. As random effects, we included intercepts for participants. The proficiency score was revealed to predict the scale of the MMN effect (*p* < 0.001, *R^2^* = 0.227) but no statistically significant result was obtained in the case of the LDN (*p* = 0.153, *R^2^* = 0.405). Further, the MMN and LDN amplitudes were both significantly predicted by the participants’ dominance operationalized in terms of the number of hours per week which they reported in the LHQ (MMN: *p* < 0.001, *R^2^* = 0.266; LDN: *p* < 0.023, *R^2^* = 0.413). Finally, a correlation was found between the scale of both ERP effects and the participants’ age of acquisition (MMN: *p* < 0.001, *R^2^* = 0.276; p, LDN: *p* < 0.001, *R^2^* = 0.422).

## 4. Discussion

The main objective of the current study was to shed more light on non-native phonological contrast perception – a phenomenon particularly relevant nowadays, with multilingualism having already become a norm in the modern globalized world (e.g., Aronin and Singleton, 2008). Previous research has demonstrated that the processing of phonological contrasts is typically hampered in non-native when compared with native languages (Jakoby et al., 2011; Song and Iverson, 2018; Liang and Chen, 2022). In the present work, we aimed to extend the scope of research in the field so that it involved two non-native languages. This way, we hoped to contribute to the ongoing discussion on the perception of native as opposed to non-native phonemes by multilingual speakers (Cabrelli and Wrembel, 2016). Specifically, we tested vowel contrast perception among L1 Polish-L2 English-L3/L*n* Norwegian speakers.

The first research question investigated whether phonological contrasts would be equally easy to detect and process in the native language (i.e., Polish) and in non-native languages (i.e., English and Norwegian). Following previous authors, we predicted that the MMN response would be stronger in native vowel perception when compared with non-native vowel perception (Näätänen et al., 1997; Jakoby et al., 2011; Song and Iverson, 2018; Liang and Chen, 2022). This hypothesis, however, was confirmed only in the case of L3/L*n* Norwegian when compared with L1 Polish. While each vowel contrast elicited a statistically significant MMN effect (Table 4), there was no statistically significant difference between the effect observed for L1 Polish and the effect elicited in L2 English (Table 6). This finding suggests that – perhaps with sufficient exposure – phonological perception mechanisms might be equally developed in the non-native language when compared with native language. Such a result is also, at least partly, in accordance with the study of Winkler et al. (1999), who found a similar MMN response to Finnish vowel contrasts in native speakers of Finnish and in naturalistic late learners of Finnish. Very importantly, however, the MMN effect observed in the current study for L3/L*n* Norwegian was statistically weaker when compared with L1 Polish. This confirms that, even for foreign languages acquired in a naturalistic setting, phonological contrasts may not always be detected as easily as in the case of one’s mother tongue.

The second research question focused on the possible emergence of any significant distinctions between L3/L*n* and L2 English. We predicted that the effect would be stronger in the more dominant and/or more proficient language. Our findings show statistically significant differences between the two foreign languages: the MMN effect was significantly stronger in L2 English when compared with L3/L*n* Norwegian. This is in accordance with our hypothesis that the effect would be enhanced for the more dominant and/or proficient foreign language. As indicated by the results of language proficiency tests, the participants in the current study – despite living in Norway – were much more proficient in English than in Norwegian. On average, they obtained 76.47% in the English proficiency test as opposed to 58.65% in the Norwegian proficiency test, and the outcomes were further supported self-assessment ratings (5.76 as opposed to 3.74 respectively). What is more, English has also turned out to be the foreign language which was more frequently used by the participants (mostly in the international work environment). Out of the 20 speakers whose data was included in the final analysis, 10 reported using English most frequently out of the three investigated languages, seven used Polish most frequently, three used English and Polish to a similar degree, but only one indicated Norwegian as their most frequently used language.

This observation is closely related to the third research question which explored the factors that might play a crucial role in L2 and L3/L*n* processing. As space does not allow for the consideration of every single one of these factors, we preliminarily distinguished AoA, proficiency, dominance and phonological aptitude as potential predictors of successful phoneme discrimination in the two non-native languages. We sought to determine whether any of these factors (measured by additional tests and self-reports) would influence the degree of the investigated ERP effects. Indeed, we found out that AoA, proficiency and language dominance impacted the MMN effect, and AoA and language dominance affected the LDN effect.In fact, the more global processing patterns reflected in the differences between the investigated language pairs (i.e., L1 vs. L2, L1 vs. L3/L*n*, L2 vs. L3/L*n*) might also enable us to point to language dominance and proficiency as two factors which seem to be of particular relevance in mastering the discrimination of non-native phonemes. This also remains in accordance with previous research on phonological discrimination mechanisms in L2 (Jakoby et al., 2011;^2,23^). However, since the results of the current study cannot fully disentangle the effects of proficiency and dominance (as the participants were apparently both more dominant and more proficient in English than in Norwegian), this distinction should be further investigated in the future.

What is also noteworthy is that the vast majority of participants started learning English in their early childhood (on average, at around the age of nine) and acquired Norwegian much later in life, well after puberty (i.e., at around 27 years of age). The measure of success in second language learning, and especially in terms of pronunciation, is frequently associated with the speaker’s age of acquisition/arrival. For example, several linguistic studies observed a positive correlation between the age of arrival to the country in which the target language is spoken and the perceived strength of accentedness (see Bongaerts et al., 1995; Flege et al., 1995, 1999, among many others). This correlation also seems to be corroborated by the current study results as reflected in the MMN difference between L2 English and L3/L*n* Norwegian.

In addition to the MMN component, deviant stimuli in all three languages have also elicited the LDN response, a component whose functional significance still remains largely unsettled. Some authors have postulated that this component reflects the pre-attentive cognitive evaluation of the stimulus, while others have associated it with the extraction of the phonological difference between the standard and deviant stimuli, the reorientation of attention to the original task, or the formation of new phonological representations (see Jakoby et al., 2011 for a discussion). In the context of non-native phoneme perception, the LDN was larger in successful compared to unsuccessful language learners (Jakoby et al., 2011) and in more advanced compared to elementary ones (Liang and Chen, 2022). These findings seem to support the last explanation proposed above, i.e., that the LDN might index a successful formation of memory traces associated with specific phonemic representations – an explanation proposed also by Barry et al. (2009). In the current study, the LDN was largest in L1 Polish, smaller in L3/L*n* Norwegian, and the smallest in L2 English, with the difference between L2 English and L3/L*n* Norwegian not statistically significant. Quite crucially, the difference between the non-native languages in question reached the level of statistical significance in the MMN time window. When interpreted together, these two findings might be viewed as tentatively supporting the idea that the LDN if functionally independent from the MMN as well as the claim that the component indexes the formation of new phonological representations (in this case, in the non-native languages). These hypotheses would need to be further verified by a longitudinal study examining the yet to established functional role of the LDN over a longer period of time.

One limitation of the current research is that – as many studies focused on multilingual language processing – it used a relatively small sample size (i.e., 20 trilingual participants). What is more, the experiment could have ideally used a mirror design, e.g., L1 Polish - > L2 English - > L3/L*n* Norwegian vs. L1 Polish - > L2 Norwegian - > L3/L*n* English (see Puig-Mayenco et al., 2020 for a discussion). Such a solution would enable us to directly compare the influence of language status on pre-attentive phonological processing and eliminate the potential confounds associated with the processing of specific vowel contrasts selected for each investigated language system. However, it would be extremely hard to find such a mirror group due to the prevalence of English as an L2 at the early stages of education. Possibly, a different combination of languages could be used in future research. In similar vein, the phonological aptitude test should ideally measure phoneme discrimination abilities in all three languages under investigation, i.e., not only in L2 English but also in L1 Polish and L3/L*n* Norwegian.

To the best of our knowledge, the current experiment was the first passive-oddball study to involve multilingual listeners. It resulted in several novel findings concerning multilingual phonological processing. Most crucially, the analysis of the ERP results revealed that the MMN was modulated by language. The MMN response in L3/*Ln* Norwegian was smaller when compared with L2 English and L1 Polish. At the same time, the LDN response in both L2 English and L3/*Ln* Norwegian was smaller when compared with L1 Polish. This provides preliminary, yet clear evidence that the foreign language status modulates auditory language processing. Living in an L3 environment does not then seem to be a guarantee of the development of native-like phonemic discrimination. Rather, it is language dominance, proficiency and age of acquisition which seem to be the most vital predictors of successful phonological difference extraction as well as the subsequent formation of new phonological representations.

## Data availability statement

The original contributions presented in the study are included in the article/Supplementary material, further inquiries can be directed to the corresponding author.

## Ethics statement

The studies involving humans were approved by Ethics Committee for Research with Human Participants at Adam Mickiewicz University. The studies were conducted in accordance with the local legislation and institutional requirements. The participants provided their written informed consent to participate in this study.

## Author contributions

HK: Conceptualization, Formal analysis, Investigation, Methodology, Visualization, Writing – original draft. KR: Conceptualization, Formal analysis, Methodology, Supervision, Visualization, Writing – review & editing. AB: Conceptualization, Methodology, Writing – review & editing. ZC: Conceptualization, Investigation, Writing – review & editing. CC: Investigation, Writing – review & editing. MW: Conceptualization, Funding acquisition, Methodology, Project administration, Supervision, Writing – review & editing.

## Footnotes

^1^There was only one participant who reported to be exposed to Norwegian before puberty, i.e., at the age of 7. The age range for the remaining 20 participants equals 20–43. It might be crucial that the early Norwegian learner has reported to be only passively exposed to Norwegian in childhood, and then started using the language at the age of 25 after moving to Norway.

^2^Jakoby et al. (2011) tested two French stimuli among Hebrew-English bilinguals, and presented formant frequencies for French stimuli, Liang and Chen (2022) tested Chinese-English bilinguals on the perception of a standard that was claimed to exist in both Chinese Mandarin and English (but which had relatively low F2 values, denoting a back, rather than a central quality of /u/). Song and Iverson (2018) used sentences. Näätänen et al. (1997) tested the perception of Estonian and Finnish vowels: they used synthetic stimuli differing in F2 only.

## References

[1] Alho K. Huotilainen M. Näätänen R. (1995). Are memory traces for simple and complex sounds located in different regions of auditory cortex? Recent MEG studies. Electroencephalogr. Clin. Neurophysiol. 44, 197–203. 7649022

[2] Archila-Suerte P. Zevin J. Bunta F. Hernandez A. E. (2012). Age of acquisition and proficiency in a second language independently influence the perception of non-native speech. Bilingualism Lang. Cogn. 15, 190–201. doi: 10.1017/S1366728911000125, PMID: 30197550 PMC6124681

[3] Aronin L. Singleton D. (2008). Multilingualism as a new linguistic dispensation. Int. J. Multiling. 5, 1–16. doi: 10.2167/ijm072.0, PMID: 17926130

[1001] Barry J. G. Hardiman M. J. Bishop D. V. (2009). Mismatch response to polysyllabic nonwords: a neurophysiological signature of language learning capacity. PLoS One. 4:e6270. doi: 10.1371/journal.pone.0006270 19609436 PMC2707009

[4] Bates D. M. Maechler M. Bolker B. (2012). lme4: linear mixed-effects models using S4 classes. R package version 0.999999-0.

[5] Bjelaković A. (2017). The vowels of contemporary RP: vowel formant measurements for BBC newsreaders. English Lang. Linguistics 21, 501–532. doi: 10.1017/S1360674316000253

[6] Boersma P. (2001). Praat, a system for doing phonetics by computer. Glot. Int. 5, 341–345.

[7] Bongaerts T. Planken B. Schils E. (1995). “Can late starters attain a native accent in a foreign language? A test of the critical period hypothesis” in The age factor in second language acquisition. eds. Singleton D. Lengyel Z. (Clevedon, GB: Multilingual Matters Limited), 30–50.

[8] Cabrelli A. J. Wrembel M. (2016). Investigating the acquisition of phonology in a third language – a state of the science and an outlook for the future. Int. J. Multiling. 13, 395–409. doi: 10.1080/14790718.2016.1217601

[9] Ceponiene R. Cheour M. Näätänen R. (1998). Interstimulus interval and auditory event-related potentials in children: evidence for multiple generators. Electroencephalogr. Clin. Neurophysiol. 108, 345–354. doi: 10.1016/S0168-5597(97)00081-6, PMID: 9714376

[10] Cohen M. X. (2014). Analyzing neural time series data: Theory and practice. Cambridge, The MIT Press.

[11] Di Dona G. Scaltritti M. Sulpizio S. (2022). Formant-invariant voice and pitch representations are pre-attentively formed from constantly varying speech and non-speech stimuli. Eur. J. Neurosci. 56, 4086–4106. doi: 10.1111/ejn.15730, PMID: 35673798 PMC9545905

[12] Díaz B. Mitterer H. Broersma M. Escara C. Sebastián-Gallés N. (2016). Variability in L2 phonemic learning originates from speech-specific capabilities: an MMN study on late bilinguals. Bilingual. Lang. Cogn. 19, 955–970. doi: 10.1017/S1366728915000450

[13] Dziubalska-Kołaczyk K. Wrembel M. (2022). “Natural growth theory of acquisition (NGTA): evidence from (mor)phonotactics” in Theoretical and practical developments in English speech assessment, research, and training. Second language learning and teaching. eds. Sardegna V. G. Jarosz A. (New York: Springer), 281–298.

[14] Flege J. E. Munro M. J. MacKay I. R. A. (1995). Factors affecting strength of perceived foreign accent in a second language. J. Acoust. 97, 3125–3134. doi: 10.1121/1.413041 7759652

[15] Flege J. E. Yeni-Komshian G. H. Liu S. (1999). Age constraints on second-language acquisition. J. Mem. Lang. 41, 78–104. doi: 10.1006/jmla.1999.2638, PMID: 35548507

[16] Hawkins S. Midgley J. (2005). Formant frequencies of RP monophthongs in four age groups of speakers. J. Int. Phon. Assoc. 35, 183–199. doi: 10.1017/S0025100305002124

[17] Jakoby H. Goldstein A. Faust M. (2011). Electrophysiological correlates of speech perception mechanisms and individual differences in second language attainment. Psychophysiology 48, 1516–1530. doi: 10.1111/j.1469-8986.2011.01227.x 21762446

[18] Jassem W. (2003). Polish. J. Int. Phon. Assoc. 33, 103–107. doi: 10.1017/S0025100303001191

[19] Kaan E. (2007). Event-related potentials and language processing: a brief overview. Lang. Linguist. Compass 1, 571–591. doi: 10.1111/j.1749-818X.2007.00037.x, PMID: 21738519

[20] Kristoffersen G. (2000). The phonology of Norwegian. Oxford: Oxford University Press.

[21] Kujala A. Näätänen R. (2003). “Auditory environment and change detection as Iindexed by the mismatch negativity (MMN)” in Detection of change. ed. Polich J. (Boston, MA: Springer)

[22] Li P. Zhang F. Yu A. Zhao X. (2020). Language history questionnaire (LHQ3): an enhanced tool for assessing multilingual experience. Bilingual. Lang. Cogn. 23, 938–944. doi: 10.1017/S1366728918001153

[23] Liang L. Chen B. (2022). The non-native phonetic perception mechanism utilized by bilinguals with different L2 proficiency levels. Int. J. Biling. 26, 368–386. doi: 10.1177/13670069211058275

[24] Luck S. J. (2005). An introduction to the event-related potential technique. Cambridge, MA: MIT Press.

[25] Luck S. J. Gaspelin N. (2017). How to get statistically significant effects in any ERP experiment (and why you shouldn’t). Psychophysiology 54, 146–157. doi: 10.1111/psyp.12639, PMID: 28000253 PMC5178877

[26] Maddieson I. (2013). Front rounded vowels. WALS Online (v2020.3), eds Dryer M. S. Haspelmath M. Z.. Available at: http://wals.info/chapter/11 (Accessed July 13, 2023).

[27] Näätänen R. Lehtokoski A. Lennes M. Cheour M. Houtilainen M. Iivonen A.. (1997). Language-specific phoneme representations revealed by electric and magnetic brain responses. Nature 385, 432–434. doi: 10.1038/385432a0, PMID: 9009189

[28] Näätänen R. Paavilainen P. Rinne T. Alho K. (2007). The mismatch negativity (MMN) in basic research of central auditory processing: a review. Clin. Neurophysiol. 118, 2544–2590. doi: 10.1016/j.clinph.2007.04.026, PMID: 17931964

[29] Oldfield R. C. (1971). The assessment and analysis of handedness: the Edinburgh inventory. Neuropsychologia 9, 97–113. doi: 10.1016/0028-3932(71)90067-4, PMID: 5146491

[30] Peirce J. W. Gray J. R. Simpson S. MacAskill M. R. Höchenberger R. Sogo H.. (2019). PsychoPy2: experiments in behavior made easy. Behav. Res. Methods 51, 195–203. doi: 10.3758/s13428-018-01193-y, PMID: 30734206 PMC6420413

[31] Peltola M. S. Kujala T. Toumainen J. Ek M. Aaltonen O. Näätänen R. (2003). Native and foreign vowel discrimination as indexed by the mismatch negativity (MMN) response. Neurosci. Lett. 352, 25–28. doi: 10.1016/j.neulet.2003.08.013, PMID: 14615041

[32] Peltola M. S. Tamminen H. Toivonen H. Kujala T. Näätänen R. (2012). Different kinds of bilinguals – different kinds of brains: the neural organisation of two languages in one brain. Brain Lang. 121, 261–266. doi: 10.1016/j.bandl.2012.03.007, PMID: 22521294

[33] Polich J. (2012). “Neuropsychology of P300” in The Oxford handbook of event-related potential components. eds. Luck S. Kappenman E. S. (Oxford: Oxford University Press), 159–188.

[34] Puig-Mayenco E. González Alonso J. Rothman J. (2020). A systematic review of transfer studies in third language acquisition. Second. Lang. Res. 36, 31–64. doi: 10.1177/0267658318809147, PMID: 36515550

[35] R Core Team (2012). R: A language and environment for statistical computing. Vienna: R Foundation for Statistical Computing.

[36] Roehm D. Bornkessel-Schlesewsky I. Rösler F. Schlesewsky M. (2007). To predict or not to predict: influences of task and strategy on the processing of semantic relations. J. Cogn. Neurosci. 19, 1259–1274. doi: 10.1162/jocn.2007.19.8.1259, PMID: 17651001

[37] Sebastian-Galles N. Baus C. (2005). “On the relationship between perception and production in L2 categories” in Twenty-first century psycholinguistics: Four cornerstones. ed. Cutler A. (London: Routledge), 279–292.

[38] Sebastián-Gallés N. Soto-Faraco S. (1999). Online processing of native and non-native phonemic contrasts in early bilinguals. Cognition 72, 111–123. doi: 10.1016/S0010-0277(99)00024-4, PMID: 10553668

[39] Slabakova R. (2017). The scalpel model of third language acquisition. Int. J. Biling. 21, 651–665. doi: 10.1177/1367006916655413

[40] Song J. Iverson P. (2018). Listening effort during speech perception enhances auditory and lexical processing for non-native listeners and accents. Cognition 179, 163–170. doi: 10.1016/j.cognition.2018.06.001, PMID: 29957515

[41] Weckwerth J. Balas A. (2019). “Selected aspects of Polish vowel formants” in Approaches to the study of sound structure and speech. eds. Wrembel M. Kiełkiewicz-Janowiak A. Gąsiorowski P. (London: Routledge), 338–348.

[42] Westergaard M. Mitrofanova N. Mykhaylyk R. Rodina Y. (2017). Crosslinguistic influence in the acquisition of a third language: the linguistic proximity model. Int. J. Biling. 21, 666–682. doi: 10.1177/1367006916648859

[43] Winkler I. Kujala T. Tiitinen H. Sivonen P. Alku P. Lehtokoski A.. (1999). Brain responses reveal the learning of foreign language phonemes. Psychophysiology 36, 638–642. doi: 10.1111/1469-8986.3650638, PMID: 10442032

[44] Wottawa J. Adda-Decker M. Isel F. (2022). Neurophysiology of non-native sound discrimination: evidence from German vowels and consonants in successive French–German bilinguals using an MMN oddball paradigm. Bilingual. Lang. Cogn. 25, 137–147. doi: 10.1017/S1366728921000468

[45] Wrembel M. (2015). In search of a new perspective: Cross-linguistic influence in the acquisition of third language phonology. Poznań: Wydawnictwo Naukowe UAM.

[46] Wrembel M. Cabrelli Amaro J. (eds.) (2018). Advances in the investigation of L3 phonological acquisition. London: Routledge.

