# Corrigendum: Vowel perception in multilingual speakers: ERP evidence from Polish, English and Norwegian

**DOI:** 10.3389/fpsyg.2024.1484655

**Published:** 2024-09-17

**Authors:** Hanna Kedzierska, Karolina Rataj, Anna Balas, Zuzanna Cal, Chloe Castle, Magdalena Wrembel

**Affiliations:** ^1^Department of Contemporary English Language, Faculty of English, Adam Mickiewicz University, Poznań, Poland; ^2^Department of English and Comparative Linguistics, Institute of English Studies, University of Wrocław, Wrocław, Poland; ^3^Neuroscience of Language Laboratory, Department of Psycholinguistic Studies, Faculty of English, Adam Mickiewicz University, Poznań, Poland; ^4^Department of Language and Culture, UiT The Arctic University of Norway, Tromsø, Norway

**Keywords:** multilingualism, speech perception, third language (L3/L*n*), event-related potentials (ERP), mismatch negativity (MMN)

In the published article, there was an error in the Funding statement. The funding institution requires a different wording for the Funding statement. The statement originally stated, “The author(s) declare financial support was received for the research, authorship, and/or publication of this article. This research is supported by Norway funds/NCN project grant GRIEG-1 (UMO-2019/34/H/HS2/00495) ADIM “Across-domain investigations in multilingualism: Modeling L3 acquisition in diverse settings”.” The corrected Funding statement is shown below.

