# Supplementary material for: Vowel perception in multilingual speakers: ERP evidence from Polish, English and Norwegian
Source: Front Psychol. 2023 Oct 12;14:1270743. doi: 10.3389/fpsyg.2023.1270743 (PMC10601651; doi:10.3389/fpsyg.2023.1270743)
Supplement: Supplementary file 1 [file Presentation_1.pdf]

Supplementary Material

Vowel perception in multilingual speakers: ERP evidence from Polish. English and Norwegian

Hanna Kędzierska*. Karolina Rataj. Anna Balas. Zuzanna Cal. Chloe Castle. Magdalena Wrembel

*** Correspondence:** Hanna Kędzierska: hanked@amu.edu.pl

1 Appendix A

This appendix contains the most relevant language history questionnaire and proficiency tests results for individual participants (Table A1). In terms of self-assessment the participants were asked to assess their skills in English and Norwegian on a scale from 1 to 7 in the following order: listening, speaking, reading, writing. By 'gating accuracy' we mean the overall percentage of words which were correctly recognized by an individual participant. By 'mean gate score' we mean the average 'gate' at which a participant recognized the word correctly (e.g., mean gate score 8.75 would indicate that on average a participant recognized the word correctly after the eighth 'gate').

Supplementary Table A1: Selected language history questionnaire and proficiency tests results for individual participants

ID code	Proficiency in English	Proficiency in Norweg.	Gating Accuracy	Mean Gate Score	Self assess. Polish	Self assess. English	Self assess. Norweg.	Years in Norway
KS7419AT	68.00%	44.44%	50.00%	8.75	7/7/7/7	6/5/6/6	3/2/3/2	2
MW5613AM	92.00%	61.11%	100.00%	7.63	7/7/7/7	7/7/7/7	4/4/4/3	6
SB5808AW	52.00%	22.22%	68.75%	7.72	7/7/7/7	6/6/7/6	N/A	3
KH7231SW	44.00%	N/A	62.50%	9.6	7/7/7/7	5/4/5/4	1/1/1/1	1
AF6315NT	92.00%	50.00%	100.00%	7.1	7/7/7/7	7/7/7/7	2/2/3/2	2

DD6822AG	84.00%	83.33%	93.75%	7.1	7/7/7/7	6/6/6/6	6/5/6/4	3
KK6310OA	88.00%	77.78%	87.50%	9.2	7/7/7/7	7/6/7/6	6/4/6/4	5
HH4419IK	100.00%	80.56%	87.50%	6.4	7/7/7/7	7/6/7/7	3/3/5/4	4
WK415AT	68.00%	36.11%	68.75%	8.1	6/6/6/5	4/4/5/4	2/1/2/2	6
MW6115AG	84.00%	94.44%	100.00%	8.3	7/7/7/7	6/6/6/6	6/6/6/6	7
SO3824OA	77.78%	76.00%	87.50%	7.2	7/7/7/7	6/5/5/5	4/4/4/4	4
WM6413OA	80.00%	75.00%	81.25%	8.9	7/7/7/7	6/6/6/5	4/4/4/4	5
RB5516NN	76.00%	94.44%	87.50%	10	7/7/7/7	5/5/6/5	6/6/6/6	12
JZ4819AS	88.00%	80.56%	75.00%	9.75	7/7/7/7	7/7/7/7	5/5/7/6	14
MM6427AR	48.00%	22.22%	62.50%	8.1	7/7/7/7	4/4/5/5	1/1/2/1	2
AA6611OM	92.00%	72.22%	62.50%	9.7	7/7/7/7	6/6/7/5	3/3/5/3	3.5
JG6428GA	96.00%	72.22%	93.75%	7.4	7/7/7/7	7/6/7/6	6/5/6/4	8
AM7023AT	72.00%	27.78%	81.25%	6.6	7/7/7/7	6/6/6/5	1/1/1/1	1
KJ6814OA	68.00%	52.78%	68.75%	7.6	7/7/7/7	5/5/6/6	5/5/6/6	7
RR7525AK	76.00%	25.00%	62.50%	8.5	7/7/7/7	5/4/6/4	4/2/2/3	1
JK5430NE	60.00%	83.33%	75.00%	9.2	7/7/7/7	6/6/5/5	5/5/4/4	4

2 Appendix B

The appendix contains figures displaying mean amplitude values (in μV) observed in each condition and each target language within the 100-200 ms time window (Figure A1) and in the 200-800 ms time window (Figure A2).

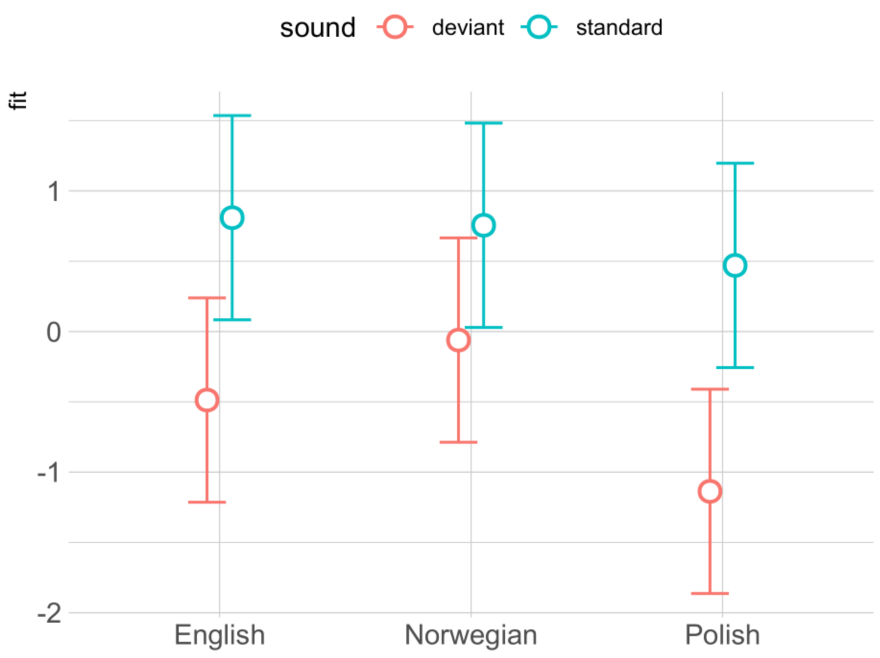

Supplementary Figure A1. Mean amplitude values (in µV) observed in each standard/deviant condition and each target language within the 100-200 ms time window

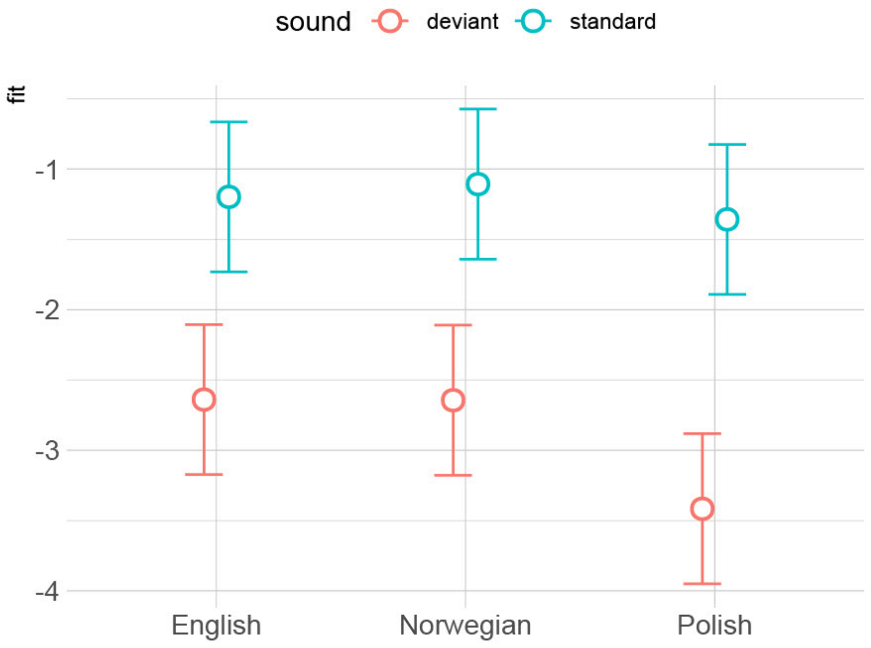

Supplementary Figure A2. Mean amplitude values (in µV) observed in each standard/deviant condition and each target language within the 350-800 ms time window

3 Appendix C

The appendix contains figures displaying mean amplitude differences (in µV) in each target language in the 100-200 ms time window (Figure A3) and in the 200-800 ms time window (Figure A4).

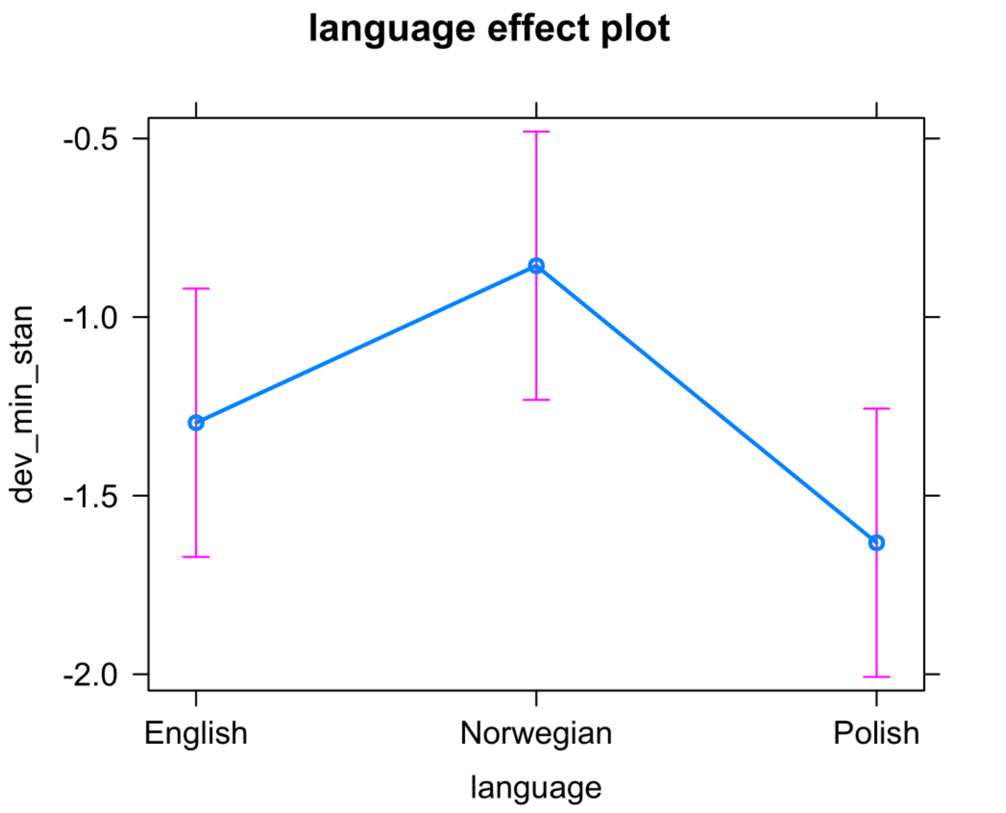

Supplementary Figure A3. Mean amplitude differences (deviant minus standard) in each target language in the 100-200 ms time window

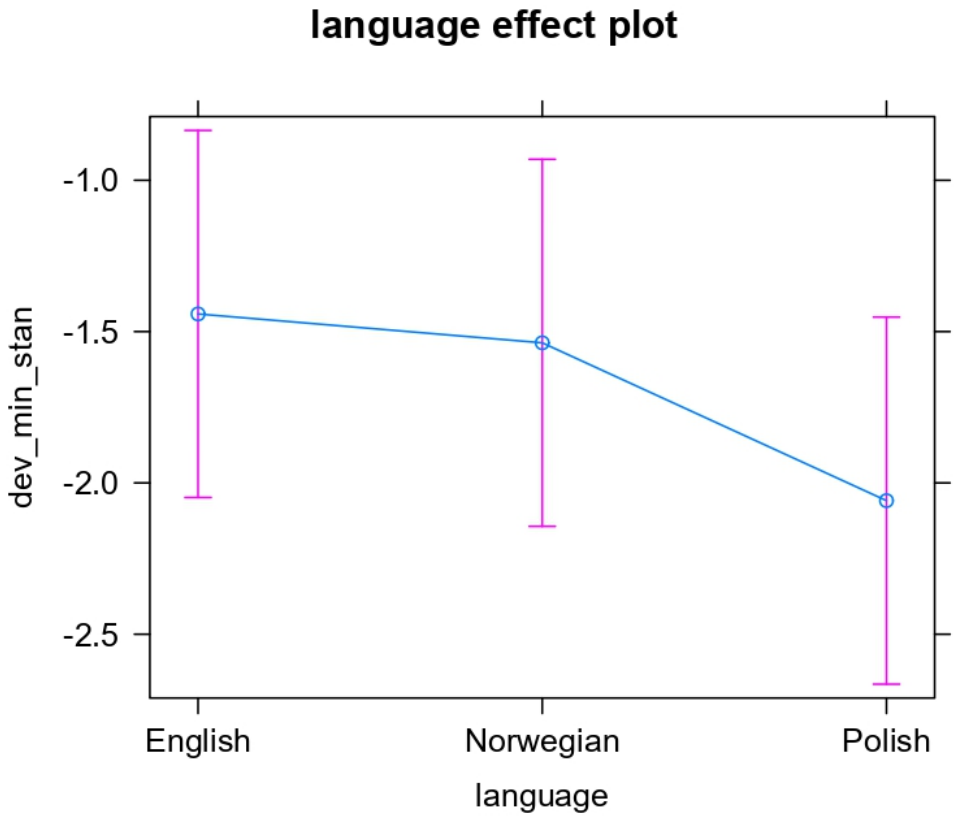

Supplementary Figure A4. Mean amplitude differences (deviant minus standard) in each target language in the 350-800 ms time window

4 Appendix D

The appendix contains figures displaying the results of correlational tests, i.e., the MMN and LDN effects versus age of arrival, dominance (associated with the number of hours per week which the participants declared to use a language) and self-assessed proficiency levels (in a 7-point scale).

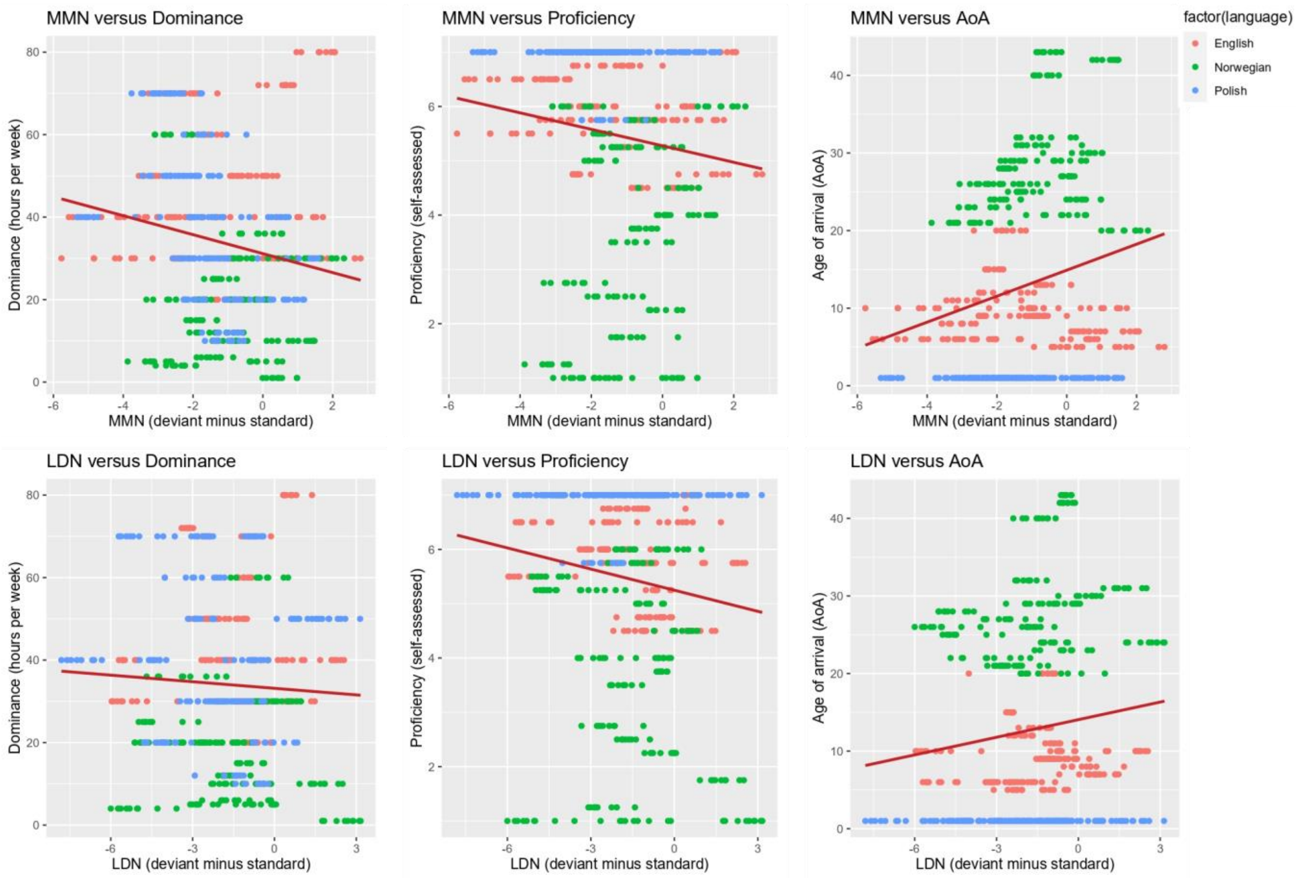

Supplementary Figure A5. The results of correlational tests assessing whether dominance, proficiency and AoA predicted the scale of MMN and LDN effects. Each point corresponds to a mean amplitude difference obtained for each participant in the MMN (100-200 ms) or LDN (350-800 ms) time window from nine representative electrodes (F3, Fz, F4, FC1, FCz, FC2, C3, Cz, C4) in three language conditions (i.e., 9 electrodes x 3 languages x 20 participants).
